# Electroconvulsive therapy modulates neurite density and free water in discrete white matter compartments in major depression

**DOI:** 10.21203/rs.3.rs-10683316/v1

**Published:** 2026-09-08

**Authors:** Artemis Zavaliangos-Petropulu, Paloma Pfeiffer, Madeline Crawford, Viviane Norris, Randall Espinoza, Pauline Wu, Jennifer Kruse, Christopher Abbott, Shawn McClintock, Yvette Sheline, Katherine Narr

**Affiliations:** University of California Los Angeles; Departments of Neurology, Psychiatry and Biobehavioral Sciences, University of California; UCLA; University of New Mexico School of Medicine; University of Pennsylvania Perelman School of Medicine; UCLA

## Abstract

Electroconvulsive therapy (ECT) is the most effective treatment for severe or intractable depression. The disrupt–potentiate–rewire framework proposes that ECT’s therapeutic effects include neural remodeling. We used the neurite orientation dispersion and density imaging (NODDI) model to characterize ECT-related longitudinal white matter (WM) microstructural changes. Fifty-two adults with treatment-resistant major depressive episodes (32 female) from two cohorts underwent multi-shell diffusion MRI and clinical assessment before the first ECT session and after completion of the index course. Plasma interleukin-6 (IL-6) was measured in one cohort. Linear mixed-effects models assessed longitudinal changes in neurite density, orientation dispersion, and free water across major WM tracts, with false-discovery-rate correction. Regression analyses examined relationships between WM changes, symptom improvement, and IL-6 change if available. Fractional anisotropy (FA) was also examined for comparison with prior studies. Depressive symptoms improved substantially, and IL-6 levels decreased following treatment. Neurite density, free water, and FA decreased across multiple frontolimbic pathways, including the uncinate fasciculus, external capsule, and internal capsule, whereas orientation dispersion remained unchanged. Free-water reductions were predominantly right-lateralized. In contrast to FA, greater NODDI-derived microstructural change was associated with greater depression symptom improvement across several mood-relevant pathways, while reductions in IL-6 showed trend-level associations with WM change. ECT was associated with coordinated reductions in neurite density and free water, alongside preserved fiber organization, within distributed WM pathways. These findings support microstructural remodeling during the putative rewiring phase of ECT and link WM change to both antidepressant improvement and changes in peripheral inflammation.

## 1. Introduction

Major depressive disorder (MDD) is a leading cause of disability worldwide[1]. Despite available pharmacological, psychotherapeutic, and neuromodulation treatments, up to a third of patients do not achieve adequate relief after multiple antidepressant treatment trials and are considered to have treatment-resistant depression (TRD)[2-4]. TRD is associated with depression relapse, functional impairment, and elevated suicide risk[4, 5]. Electroconvulsive therapy (ECT) is the most effective FDA-cleared treatment available for TRD[6-8] as well as for severe depressive and psychotic conditions requiring rapid response[9]. With ECT, a brief electrical stimulus is delivered under anesthesia to induce a generalized seizure that engages widespread cortical and subcortical networks, producing marked antidepressant improvement in 70–90% of patients, and remission in over half, well above pharmacotherapy outcomes[10, 11]. Yet, after more than eight decades of clinical use, ECTs therapeutic mechanisms require clarification. The "disrupt, potentiate, rewire" hypothesis offers one potential mechanistic framework of ECT[12]: the stimulus first disrupts pathological network activity, then potentiates neuroplasticity at the molecular and cellular level, and ultimately rewires large-scale functional networks to support antidepressant response.

Diffusion MRI (dMRI) offers a noninvasive window into ECT’s “rewire” component by measuring white matter (WM) microstructure and connectivity, potentially informing treatment mechanisms and identifying response biomarkers[13]. However, using the diffusion tensor imaging (DTI) model exclusively, studies reporting ECT-related changes in WM have been inconsistent regarding direction and regional distribution of effects. For example, fractional anisotropy (FA) has been reported to increase[14, 15], decrease[16, 17], or show no significant change[18, 19] following ECT. Mean diffusivity (MD), which represents the overall magnitude of diffusion across all directions, has yielded somewhat more reproducible findings, with studies generally reporting increases in MD following ECT[14, 16, 18].

Notably, the DTI model assumes a single dominant fiber orientation per voxel and thus fails to capture the complexity of neural architecture[20]. Specifically, though indexing general WM organization[21], FA conflates intracellular and extracellular diffusion and free-water contamination into a single composite, thus obscuring microstructural measures associated with specific neural processes. Biologically informed multi-compartment models such as neurite orientation dispersion and density imaging (NODDI)[22] address this limitation by modeling intra-neurite, extra-neurite, and free-water compartments separately. From these modeled compartments, NODDI derives three complementary measures of tissue microstructure. Neurites are the projections extending from neural cells, including both axons and dendrites. The neurite density index (NDI) estimates the fraction of tissue within a voxel occupied by neurites, providing a measure of their relative density. The orientation dispersion index (ODI) quantifies how coherently neurites are oriented within a voxel, distinguishing regions with highly aligned fibers from those with more dispersed fiber organization. The free-water fraction (FISO) estimates the proportion of freely diffusing extracellular water, providing information about the extracellular tissue environment. By resolving these compartments, multi-shell diffusion MRI can detect microstructural changes the tensor model cannot, including reorganization in tissue architecture that may clarify ECT’s antidepressant mechanisms. Though a prior ECT study used NODDI to examine gray matter tissue ECT[23], no prior investigation has applied biologically informed non-tensor models to measure WM changes, leaving a gap in understanding of the microstructural basis of ECTs effects on WM connectivity.

Converging evidence indicates that changes in brain macro- or microstructure occurring with ECT reflect neuroplastic remodeling rather than tissue injury with no evidence of structural brain damage reported[24-27]. However, transiently, ECT can produce an acute increase in free water in periventricular and subcortical regions[28], which align with observations of a brief neuroinflammatory or neurogenic response that resolves over the index treatment course[29]. These observations also parallel the reductions in peripheral inflammatory markers reported after successful ECT[30, 31], suggesting that its longer-term effects may include an anti-inflammatory component. Within the NODDI framework, FISO is well-suited to tracking this transient process and its resolution, complementing NDI and ODI as indices of more stable WM reorganization.

To clarify the effect of ECT on WM remodeling, we applied multi-shell diffusion MRI with NODDI in two independent ECT cohorts recruited at the University of California, Los Angeles (UCLA), quantifying longitudinal change in NDI, ODI, and FISO using tract-based spatial statistics (TBSS) and atlas-defined WM pathways. We hypothesized that ECT would produce NDI and ODI remodeling in frontotemporal and limbic pathways implicated in mood regulation. Given prior evidence for a transient neuroinflammatory response that resolves with continued treatment[32-34], we further predicted that FISO would decrease across the index series, reflecting normalization of extracellular water. We further examined whether microstructural changes were associated with improvement in depressive symptoms and, in one cohort, with change in peripheral interleukin-6 (IL-6).

## 2. Methods

### 2.1 Study Design

Data were drawn from two independent electroconvulsive therapy (ECT) studies conducted at UCLA: (1) the Deciphering Mechanisms of ECT Outcomes and Adverse Effects (DECODE; R01 MH128690), a multi-site study including UCLA, the University of New Mexico, the University of Texas Southwestern Medical Center, and the University of Pennsylvania, designed to identify neural and cognitive predictors of ECT response associated with dose measured with individualized electric-field modeling, and (2) the Human Connectome Project—Connectomes Related to Human Disease (HCP-CRHD; U01 MH110008), which applied advanced multimodal neuroimaging to characterize neurobiological changes associated with ECT (NCT02165449) and other antidepressant treatments. The DECODE study is ongoing, with data collection anticipated to continue through 2027. Because only the UCLA site had more than ten participants with longitudinal dMRI at the data freeze (May 2026), analyses were restricted to UCLA participants to avoid unstable estimation of site effects.

For both HCP-CRHD and DECODE, the clinician-rated Inventory of Depressive Symptomatology (IDS-C) [35] was used as the primary measure of antidepressant outcome and was acquired at two time points: within 24 hours before the first ECT session (baseline) and within one week after completion of the ECT index series (post-index)(Fig. 1).

### 2.2 Participants

Patients participating in one of two clinical research studies (HCP-CRHD and DECODE) had largely overlapping inclusion and exclusion criteria Participants met DSM-5 criteria for a moderate-to-severe major depressive episode based on semi-structured diagnostic interviews[36, 37] and had TRD, defined as persistent depression despite at least two adequate antidepressant trials[2, 3].

Eligible participants were adults (≥ 20 years) deemed appropriate candidates for ECT following standard clinical evaluation by the Resnick Neuropsychiatric Hospital at UCLA (R-NPH) ECT team. Exclusion criteria included serious medical or neurological illness, substance use disorder within the past 3 months, schizophrenia or schizoaffective disorder, psychosis secondary to a medical condition, history of convulsions or withdrawal seizures, rapid-cycling bipolar disorder, or neuromodulation or ketamine treatment within the previous 3 months. Additional research eligibility criteria included English fluency, no MRI contraindications, and willingness to share de-identified data in NIH research repositories. Participants enrolled in the HCP-CRHD study were also required to discontinue antidepressant and anticonvulsant medications prior to initiating ECT treatment. DECODE participants were not required to discontinue psychotropic medications; however, anticonvulsants, benzodiazepines, and other medications known to affect seizure threshold were tapered or withheld in accordance with the clinical ECT protocol.

All participants provided written informed consent before study procedures. Study protocols were approved by the UCLA Institutional Review Board and, for DECODE participants, the University of Pennsylvania Institutional Review Board serving as the single IRB. Clinical and demographic characteristics of the study sample are summarized in Table 1.

### 2.3 ECT Treatment

Both studies used a naturalistic design. ECT was administered three times weekly using a MECTA SIGMA device (MECTA Corp., Tualatin, OR, USA) with seizure threshold (ST) titration. Anesthesia and muscle relaxation were achieved with methohexital (1 mg/kg) and succinylcholine (1 mg/kg), respectively.

For the DECODE cohort, ECT procedures followed a predefined treatment algorithm. Participants initiated treatment with right unilateral (RUL) brief-pulse (0.5 ms) ECT, with the exception of one participant who received bifrontal (BF) placement throughout the index course. Subsequent RUL treatments were administered at six times the seizure threshold. Participants with suboptimal early response, defined as less than a 25% reduction in depressive symptoms on the IDS-C[38] by the sixth ECT treatment were switched to standard bitemporal (BT) brief-pulse (0.5 ms) ECT beginning with the seventh treatment. Following stimulus dose titration, subsequent BT treatments were administered at two times the seizure threshold.

Participants in the HCP-CRHD cohort also initiated treatment with RUL ECT, using an ultrabrief pulse width (0.3 ms) at five times the seizure threshold. Based on clinical judgment of treatment response, participants could be switched to bitemporal ECT, delivered with a brief pulse width (0.5 ms) at 1.5 times the seizure threshold, at any point during the index ECT series. Two participants were switched to left anterior right temporal (LART) electrode placement as clinically indicated.

### 2.4 Peripheral biomarker assays

For the HCP-CRHD dataset only, venous blood samples were also collected at baseline and post-treatment to enable measurement of peripheral inflammatory markers. IL-6 levels were measured using a Meso Scale Discovery (MSD) electrochemiluminescence assay. Assays were performed in duplicate, and concentrations were quantified using a standard curve. The lower limit of detection for IL-6 was 0.2 pg/mL, with all samples above the detection threshold. Mean intra-assay and inter-assay CVs were 2.7% and 3.4%, respectively, demonstrating excellent reproducibility. These measures were used to examine associations between dMRI indices and systemic inflammatory changes across treatment in the HCP-CRHD cohort exclusively.

### 2.5 MRI acquisition

MRI data were acquired at the UCLA Ahmanson-Lovelace Brain Mapping Center on a 3T Siemens Prisma scanner using a 32-channel head coil. HCP-CRHD participants were scanned using Human Connectome Project (HCP) Lifespan protocols[39], whereas DECODE participants were scanned using a protocol incorporating sequences from the HCP Lifespan projects and the Adolescent Brain Cognitive Development (ABCD) Study[40].

All participants underwent high-resolution T1-weighted (T1w) MPRAGE and T2-weighted (T2w) SPACE structural imaging (0.8-mm isotropic resolution). T2w images were co-registered to T1w images to improve gray-white matter boundary delineation for cortical and subcortical segmentation.

Diffusion MRI (dMRI) was acquired using multi-shell, multiband high-angular-resolution diffusion imaging (HARDI). HCP-CRHD participants were scanned using the HCP Lifespan diffusion protocol (1.5-mm isotropic resolution; b = 1500 and 3000 s/mm^2^), whereas DECODE participants were scanned using a modified ABCD protocol (2.0-mm isotropic resolution; b = 500, 1000, 2000, and 3000 s/mm^2^). Both protocols included opposing phase-encoding directions for susceptibility distortion correction. Complete acquisition parameters are provided in **Supplemental Table 1**.

### 2.6 Diffusion MRI processing

All MRI data were preprocessed using the Human Connectome Project (HCP) minimal preprocessing pipeline[41], adapted for the two multi-shell diffusion datasets. Processing steps implemented in FSL[42] included normalization of b0 intensities across runs, correction for eddy-current and susceptibility-induced distortions, adjustment for head motion and gradient nonlinearities, and registration of diffusion-weighted volumes to the corresponding T1-weighted anatomical image. Each diffusion dataset was visually inspected for motion artifacts and signal dropout.

After quality control, DTI and NODDI models were fitted separately to the data. NODDI modeling was performed using the Dmipy toolbox[43], which implements a multi-tissue adaptation of the NODDI framework based on a response function estimated through constrained spherical deconvolution. This model yields three biologically interpretable metrics: NDI, ODI, and FISO. Each parameter map was weighted by the WM volume fraction derived from the multi-tissue response to isolate the WM signal contribution. Diffusion tensor fitting was performed using FSL’s *DTIFIT*[44, 45] to generate fractional anisotropy (FA) maps for reference. FA was analyzed and included as a supplementary metric. TBSS[46] were applied following a longitudinal protocol adapted from Engvig et al. (2012)[47], previously described[48].

All analyses were conducted using a tract ROI-based approach focused on major WM tracts defined by the Johns Hopkins University (JHU) ICBM-DTI-81 White Matter Atlas[49]. The atlas, thresholded at 25% probability, includes 38 hemispheric WM fiber pathways (**Supplemental Table 2**). Mean values of NDI, ODI, FISO, and FA were extracted from the skeletonized maps for each ROI and time point. ROI masks were applied to the normalized, skeletonized NODDI and DTI maps in standard space using FSL’s *fslstats* tool[45].

### 2.7 Statistical Analyses

All statistical analyses were conducted in R (v4.3). Analyses were performed separately within each dataset (HCP-CRHD and DECODE) and repeated in the pooled sample, with *study* included as a fixed effect to account for study-level variance. False discovery rate (FDR) correction using the Benjamini–Hochberg procedure[50] (*q* < 0.05) was applied to adjust for multiple comparisons across WM regions and diffusion metrics.

#### 2.7.1 Depressive Symptoms

To evaluate the overall antidepressant effect of ECT, paired t-tests compared baseline and post-index IDS-C total scores[36]. To examine symptom-domain changes, we calculated anxiety (IDS-C_anxiety_; items 7, 25, 26–28) and core depression (IDS-C_depression_; items 5, 15, 16, 19, 20) subscales, each ranging from 0–15, with scores ≥ 7 indicating clinically significant symptoms. Paired t-tests were then used to compare baseline and post-index subscale scores within each dataset and in the pooled sample.

#### 2.7.2 Cytokine Levels

In the HCP-CRHD cohort, paired t-tests were used to examine changes in plasma cytokine concentrations (IL-6) between baseline and post-index assessments. These analyses tested whether ECT was associated with changes in peripheral inflammation. We also tested for correlations between change in IL-6 with change in IDS-C.

#### 2.7.3 White Matter Microstructure

Treatment-related changes in diffusion metrics were assessed using linear mixed-effects models examining the effect of time on NDI, ODI, FISO, and FA metrics extracted from JHU WM tract ROIs. Models included time, age, sex, and study (DECODE or HCP-CRHD) as fixed effects and participant as a random effect. To evaluate the influence of ECT electrode placement, we calculated the proportion of right unilateral (RUL) sessions for each participant and tested a Time*%RUL interaction. This model was compared with the primary model using likelihood-ratio tests, ΔAIC, and changes in the time-effect coefficient for each ROI.

#### 2.7.4 Associations with Clinical Improvement

To determine whether imaging changes were associated with symptom improvement, linear regression models were used to examine the relationship between changes in diffusion metrics (ΔROI=post-index–baseline) and percent changes in IDS-C scores (ΔIDS=(post-index–baseline)/baseline). In each model, ΔIDS served as the dependent variable and ΔROI as the predictor of interest, with age and sex included as covariates. These analyses were repeated using the IDS-C_anxiety_ and IDS-C_depression_ subscales.

#### 2.7.5 Associations with inflammatory response

Within the HCP-CRHD cohort, multiple linear regression was used to test whether percent change in IL-6 predicted changes in diffusion metrics within each tract ROI. Models adjusted for age, sex, and baseline ROI values to account for regression to the mean.

## 3. Results

### 3.1 Participants

A total of 52 participants were included across both studies (61.5% female, age = 40 ± 15.7 years). Additional demographics and a breakdown by study are presented in Table 1.

### 3.2 Change in Depressive Symptoms

IDS-C total (t(51) = − 9.02) and IDS-C_depression_ (t(51) = − 9.60) decreased markedly from baseline to post-index in the pooled sample (both p < 0.001), with significant reductions in each cohort individually (all p < 0.001; Fig. 2A, 2C). IDS-C_anxiety_ showed a smaller but significant decrease (t(51) = − 3.40, p = 0.001; Fig. 2D), driven primarily by DECODE (t(23) = − 4.48, p < 0.001), with no significant change in HCP-CRHD alone (p > 0.05).

### 3.3 Change in Cytokine Levels

IL-6 significantly decreased from baseline to post-index in the HCP-CRHD dataset (t(26) = − 2.37, p = 0.026; Fig. 2B). Percent change in IL-6 was not associated with percent change in IDS-C total or subscales (p > 0.05) over treatment.

### 3.4 Change in White Matter Microstructure

Across all metrics, significant changes reflected decreases from baseline to post-index (Table 2, Fig. 3). NDI showed 2 FDR-significant regions and 8 trend-level findings. FISO showed 3 FDR-significant and 5 trend-level regions, primarily in the right hemisphere. ODI showed no FDR-significant changes but 3 trend-level decreases. FA demonstrated the most widespread effects, with 11 FDR-significant and 5 trend-level regions **(Supplemental Table 4, Supplemental Fig. 1**). Overall, these findings indicate widespread reductions in NDI, FISO, and FA following the index ECT series.

As a post-hoc sensitivity analysis, we added a time×percent RUL interaction to the model to test whether the proportion of RUL sessions by index altered regional WM effects. The interaction reached p < 0.05 by likelihood-ratio test in 12/152 ROI×metric tests (~ 8%), only modestly above the 5% null rate.

### 3.5 Correlations with change in mood

#### IDS-C total

Greater improvement in IDS-C total was associated with decreases in NDI in the right uncinate fasciculus, superior longitudinal fasciculus, and anterior corona radiata, and with increases in the right cingulum and left external capsule. For FISO, improvement was associated with decreases in the right posterior, superior, and anterior corona radiata, right superior longitudinal fasciculus, and left fornix-crest/stria terminalis, and with increases in the left external capsule. Although ODI showed no significant effect of time, clinical improvement was associated with decreased ODI in the left posterior limb of the internal capsule and increased ODI in the fornix column and body and right retrolenticular internal capsule. No FA association reached significance.

#### IDS-C_depression_

Improvement was associated with increased NDI in the left external capsule, decreased ODI in the left posterior limb of the internal capsule, and increased ODI in the fornix column and body. No FA or FISO association reached significance.

#### IDS-C_anxiety_

Improvement was associated with decreased NDI in the right corticospinal tract and increased NDI in the right external capsule and right tapetum, decreased ODI in the right fornix-crest/stria terminalis, decreased FISO in the left fornix-crest/stria terminalis, and decreased FA in the left and right hippocampal cingulum

Effect sizes and p-values for all associations are reported in **Supplemental Table 5**. Summary maps are shown in Fig. 4, with individual scatter plots in **Supplemental Figs. 2–4**.

### 3.6 Correlations with IL6

Larger decreases in IL-6 tracked with changes in diffusion metrics at the uncorrected level (p < 0.065) only, and included decreases in FISO of the right uncinate fasciculus (p = 0.064, r = 0.38) and NDI of the left posterior corona radiata (p = 0.061, r = 0.39). Decreases in IL-6 also tracked with increases in ODI in the right superior fronto-occipital fasciculus (p = 0.026, r=−0.45) and FA of the left fornix/stria terminalis (p = 0.015, r = − 0.49), the left superior fronto-occipital fasciculus (p = 0.029, r = − 0.45), the right inferior fronto-occipital fasciculus (p = 0.042, r = − 0.42), and the right cingulum (p = 0.057, r = − 0.39) (Fig. 5).

## 4. Discussion

To investigate the modulation and neural rewiring of the WM connectome with ECT, we applied multi-shell NODDI imaging in two prospective cohorts of patients with major depression undergoing a clinically prescribed course of ECT. To our knowledge, this is the first study to use multi-shell NODDI to characterize longitudinal changes in specific WM compartments during ECT, thereby extending prior observations of ECT-associated WM plasticity using single-shell acquisition and DTI models[13]. We additionally examined whether changes in WM diffusion measures were associated with symptom improvement and with change in peripheral IL-6, both as a test of their potential utility as biomarkers of antidepressant response and as an initial probe of the biological processes accompanying treatment.

Consistent with the established efficacy of ECT in major depression[6, 51, 52], patients showed substantial improvement in depressive and anxiety symptoms over the index course, alongside significant reductions in peripheral IL-6[29]. NDI and FISO decreased across multiple major WM pathways pre-to-post ECT, with changes concentrated in corticolimbic tracts. FA decreased across an overlapping set of tracts, while ODI showed no significant change following FDR correction. Changes in NDI and FISO were associated with symptom improvement in several pathways, in both directions depending on the tract’s location, whereas changes in FA were not associated with symptom change in any pathway. At the uncorrected level, decreases in NDI and FISO, together with increases in FA, were associated with decreased IL-6 in limbic (uncinate) and subcortical projection (corona radiata) pathways.

### 4.1. ECT modulates white matter microstructure across multiple depression-relevant white matter pathways.

Our findings indicated pronounced changes in NODDI metrics of NDI and FISO, as well as of FA, in multiple major WM pathways following ECT that align with the proposed "disrupt, potentiate, rewire" mechanistic framework of ECT[12]. Specifically, seizure-induced perturbation of established neural patterns appear to engage plasticity-related processes that culminate in microstructural remodeling. The observed decreases in NDI, together with changes in FISO and FA within tracts implicated in depression and ECT response, are compatible with this framework and may reflect plasticity-related reorganization of these pathways over the course of treatment, though noting other mechanistic accounts remain possible.

At the spatial level, the observed changes appeared concentrated in tracts implicated in both depression and ECT. The uncinate fasciculus and cingulum are central components of cortico-limbic models of mood disorders[12, 53, 54], whereas the fornix and stria terminalis serve hippocampus and amygdala structures that reliably show change following ECT[55-58]. Several affected pathways, including the uncinate fasciculus, inferior fronto-occipital fasciculus, and internal capsule, have also been implicated in previous diffusion studies of ECT[16, 18]. This convergence across studies suggests that ECT-related WM changes are concentrated within neural systems engaged by treatment and relevant to depressive illness, rather than being randomly distributed throughout the brain.

### 4.2 ECT-related changes of neurite density, free water and FA

Because traditional DTI metrics such as FA reflect multiple biological processes, including axonal density, myelination, fiber coherence, extracellular water, inflammation, and crossing fibers, analyzing distinct cellular and extracellular compartments provides greater mechanistic insight into the neurobiological effects of ECT. Since increased free water generally lowers apparent anisotropy[59], a decrease in FISO would be expected to increase FA. However, here FA decreased alongside FISO, indicating that the observed FA reductions cannot be explained solely by changes in extracellular free water. ODI showed no significant change after FDR correction, likewise, indicating that the reductions in FA and NDI were not accompanied by detectable alterations in fiber dispersion. Together, these findings point toward change within the tissue compartment rather than extracellularly. Acknowledging that in-vivo diffusion imaging-derived metrics cannot directly probe and quantify neuroplastic changes at the molecular and cellular level[60], our results infer that ECT elicits alterations in neurite architecture, glial processes, axonal packing, or other forms of structural plasticity previously associated with ECT[52, 61]. Notably, the only prior NODDI study in ECT, which examined changes in gray matter properties in the hippocampus rather than in WM, reported a similar pattern of reduced neurite density with preserved dispersion and likewise interpreted these findings as compatible with adaptive tissue remodeling[17].

Prior studies of ECT that relied on single-shell diffusion tensor imaging have produced mixed results[13]. However, several studies have reported ECT-related increases in MD, which have been interpreted to reflect increases in extracellular water potentially associated with transient blood-brain barrier permeability or inflammation[16, 17, 62]. The basis of interpreting increases in MD or free water has been partly inherited from neurodegenerative and aging research, in which diffusion metrics are treated as indices of tissue health: lower FA or NDI as reduced "WM integrity," and higher MD or FISO as a marker of pathology[63-66]. However, these metrics are context dependent, and no single metric is interpretable in isolation.

The concurrent reductions in NDI and FISO observed here provide a more nuanced explanation for previously reported ECT-related diffusion findings from single-shell data. Because each NODDI metric can reflect multiple underlying biological processes, interpretation depends on the pattern across intracellular, extracellular, and free-water compartments rather than any single measure in isolation. In the present study, FISO and NDI decreased while orientation dispersion was preserved, inconsistent with neurodegenerative or edematous processes typically associated with increased free water. In a treatment setting characterized by substantial clinical improvement and evidence of neuroplastic change[51, 61], reductions in NDI with concurrent reduction in FISO suggests that persistent extracellular fluid accumulation is unlikely to be the primary driver of the post-treatment diffusion changes observed in this cohort. Instead, the findings are more consistent with microstructural alterations occurring within the tissue compartment.

### 4.3 Relationships with clinical outcomes

In line with the well-documented clinical benefits of ECT [11, 51], patients showed substantial improvements in overall IDS-C and IDS-C_depression_ and IDS-C_anxiety_ over the index course. Changes in NDI and FISO were associated with clinical improvement across several pathways linking cortical and limbic regions, though not uniformly in direction. This bidirectionality was spatially organized rather than diffuse: associations in which greater reductions accompanied improvement were concentrated in right-hemisphere association and projection tracts for both metrics, whereas greater increases in both NDI and FISO within the left external capsule were associated with greater improvement, an association also observed for the core depression subscale. The convergence of NDI and FISO findings in the left external capsule suggests this region may represent a particularly robust locus of treatment-related microstructural change. Though the internal capsule was not included as a JHU-atlas ROI, the focus of prior deep brain stimulation (DBS) trials on targeting the anterior limb of the internal capsule/ventral capsule and ventral striatum in depression proximal to the external capsule also connecting prefrontal–striatal–thalamic regions involved in mood and reward align with this observation[67].

Taken together, these findings support a pattern of tract- and hemisphere-specific WM reorganization associated with antidepressant response. Reductions in NDI and FISO across predominantly right-lateralized pathways coexisted with increases within the left external capsule, suggesting that ECT-related microstructural remodeling is regionally heterogeneous rather than uniformly directional. Whether this right-lateralization is influenced by the predominant use of right unilateral electrode placement, is unclear. Although sensitivity analysis found no clear effect of RUL proportion, electrode-placement heterogeneity limited power for comparing RUL vs. BT, leaving a stimulation-related contribution unresolved and warranting future individualized electric-field modeling. Although provisional, these findings are consistent with a model in which treatment induces compartment-specific changes in neurite density and extracellular free-water content, the magnitude of which scales with clinical improvement. The observed bidirectionality may also reflect different modulation of WM tracts connecting different functional networks or stages of the response process, with distinct microstructural changes predominating over the course of treatment.

ODI associations also emerged in mixed directions despite no overall effect of time. Because ODI reflects neurite dispersion, these findings further track with the notion that regional changes in fiber architecture contribute to ECT’s therapeutic mechanism of action but require replication. By contrast, FA changes were not associated with clinical improvement, likely reflecting its limited biological specificity. Prior diffusion studies, conducted almost exclusively using single-shell tensor imaging, have similarly failed to identify consistent relationships between ECT-related WM change and clinical response[16, 18, 19]. That symptom associations emerged in compartment-specific NODDI metrics but not FA suggests, pending replication, that multi-shell approaches may provide the specificity needed to detect clinically relevant WM remodeling.

### 4.4 Relationships with inflammatory response

Peripheral IL-6 decreased over the index course, consistent with previous reports that inflammatory markers decline after ECT[30, 32, 68]. In this subsample, changes in IL-6 were associated with increases in FA and decreases in NDI and FISO, though only at the uncorrected level. Given that peripheral markers and central nervous system processes only partially overlap[69], these findings tentatively suggest that ECT-related WM change may be modulated by inflammatory activity. The NDI and FISO effects are of greater interest than FA, which lacks biological specificity, given proposed links between ECT, neuroinflammation, and the disrupt, potentiate, rewire model[12]. Using restriction spectrum imaging (RSI) before and after a single ECT session, Takamiya et al. reported an acute increase in free-water-related diffusion with decreased restricted diffusion, interpreted as transient vasogenic edema rather than cellular injury and resolving over subsequent weeks[28]. This parallels our prior work showing that ECT induces a transient inflammatory spike that returns to baseline or below by the end of treatment[32-34]. IL-6 was sampled only at baseline and after the index course, capturing inflammatory change at the later stage of treatment. Although the compartments estimated by RSI and NODDI are not directly equivalent, the observed decrease in FISO suggests that any early free-water increase had declined by the end of the course, with no persistent extracellular fluid accumulation. Longitudinal peripheral and central inflammatory measures alongside advanced diffusion imaging will be needed to clarify whether immune changes contribute to ECT-related WM alterations.

### 4.5. Limitations

Several limitations should be considered. Most importantly, our study was naturalistic, and the two-timepoint design, with scans acquired at baseline and after the index course, does not capture any possible trajectory of change between timepoints. Secondly, NODDI metrics are model-derived estimates of tissue microstructure rather than direct measures of specific cellular processes[22, 70, 71]. Although they provide greater compartmental specificity than conventional diffusion tensor imaging, the biological basis of changes in NODDI metrics remain indirect. Reliable estimation of the NODDI compartments also optimally require multi-shell acquisition;[72]. Multi-shell protocols are longer and more demanding and have only recently become more commonly adopted, which limits the pooling of archival data for large-scale retrospective or harmonized analyses.

Although we leveraged a within-subjects design, our sample was modest, limiting power for subgroup analyses. Replication of the current findings with the full sample is planned at DECODE study completion. We did not measure central neuroinflammation or blood-brain barrier function directly; the symptom and IL-6 correlations should therefore be considered exploratory. Finally, we lacked a large healthy control group for direct test-retest comparison. However, we have previously demonstrated that these diffusion metrics are stable across repeated scans in the absence of intervention[48], making measurement drift an unlikely explanation for the observed changes.

### 4.6. Conclusion

Across the index course, ECT was associated with widespread reductions in FA, NDI, and FISO, concentrated in corticolimbic pathways relevant to depression and to ECT. Depressive symptoms and peripheral IL-6 also decreased over the same period. By separating the tissue and free-water compartments, NODDI offered greater specificity than conventional tensor imaging and revealed a coordinated pattern: reduced neurite density and free water in tracts whose fiber organization remained intact, which we interpret as microstructural remodeling during the reorganizational phase of ECT. Changes in particular pathways were further associated with antidepressant and anxiolytic improvement and with reductions in inflammatory signaling. Denser longitudinal sampling and complementary biological measures will be needed to map the time course of these changes and identify the cellular processes underlying them.

## Supplementary Material

This is a list of supplementary files associated with this preprint. Click to download.

• SupplementalMaterial.docx

## Figures and Tables

**Figure 1:**
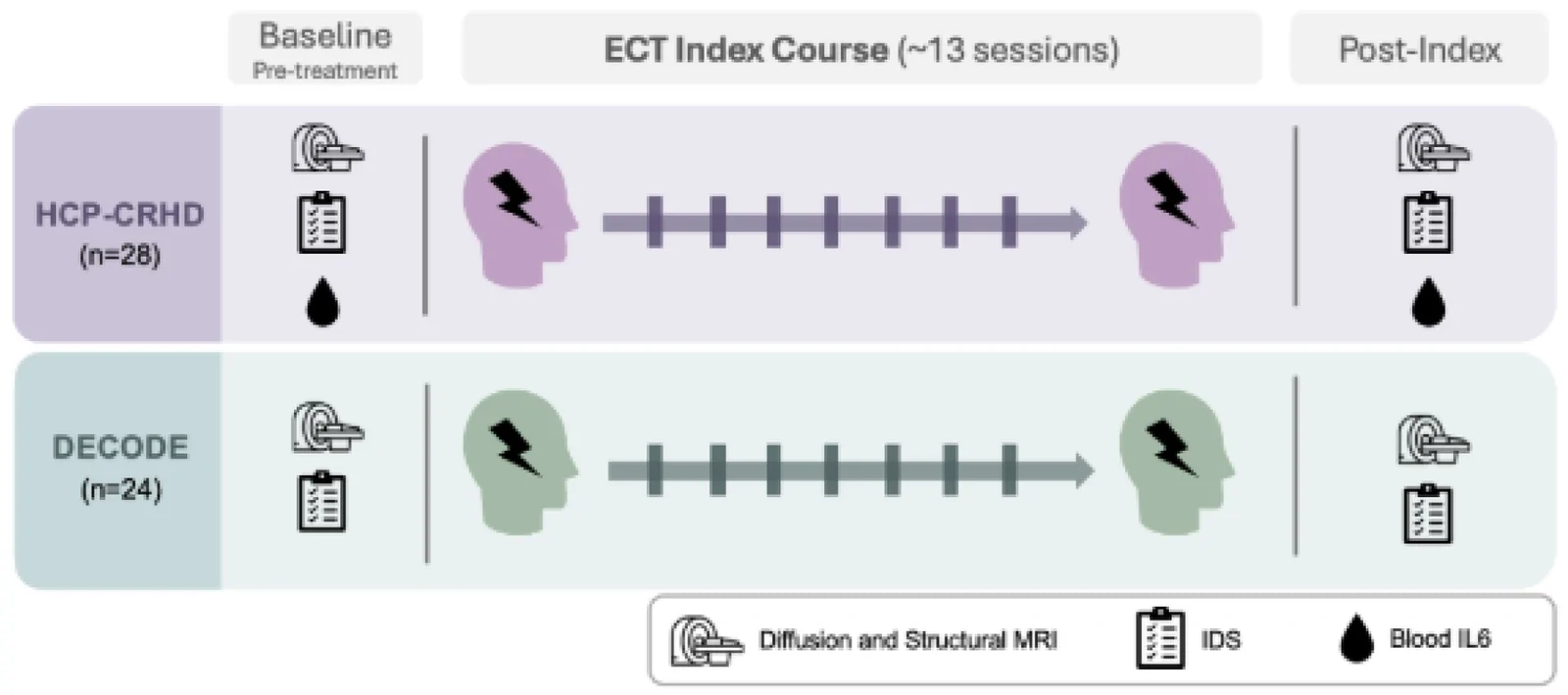
Schematic of study design including two longitudinal ECT cohorts, DECODE (n = 24) and HCP-CRHD (n = 28), where multi-shell diffusion MRI and IDS-C depression ratings were collected at Baseline and Post-index (~13 sessions apart); HCP-CRHD also collected plasma IL-6 at both time points. Both studies began with right unilateral (RUL) electrode placement, with the exception of one DECODE participant who received bifrontal (BF) ECT throughout the index course: For DECODE, patients with <25% change in mood ratings were switched to bitemporal (BT) ECT after the 6th treatment, while for HCP-CRHD patients were transitioned to BT and two participants were switched to left anterior right temporal (LART) electrode placement, a specialized, asymmetric bilateral technique, based on clinical grounds.

**Figure 2:**
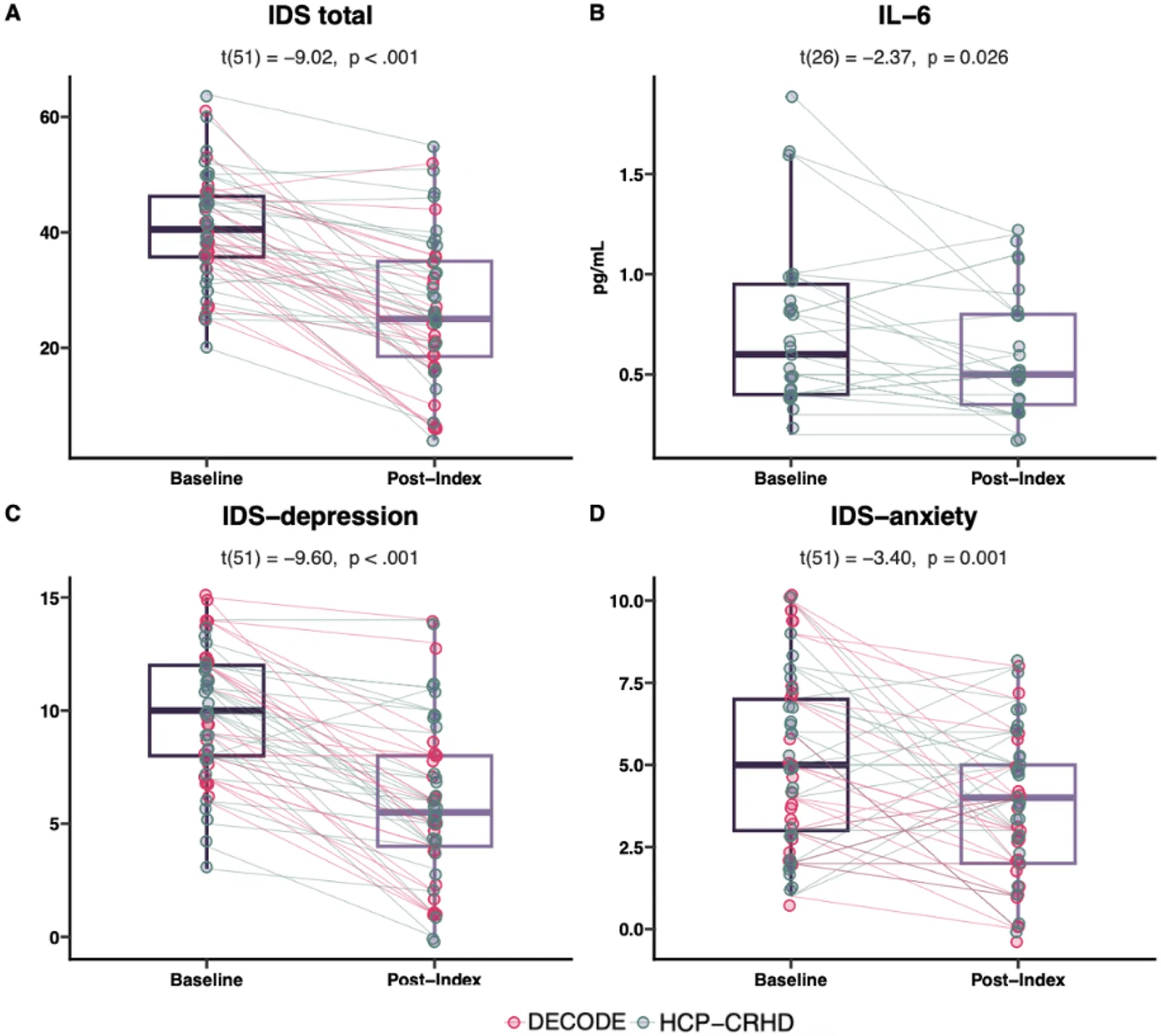
Improvements in depressive symptoms and reductions in plasma IL-6 across ECT. Box-and-whisker plots show (A) IDS-C total score (lower = less depression), (B) plasma IL-6 in pg/mL (lower = reduced inflammation), (C) IDS-C core depression subscale, and (D) IDS-C anxiety subscale at baseline and post-index. All four measures decreased significantly across treatment (paired-samples t-tests, statistics above each panel). Points are colored by the DECODE and HCP-CRHD ECT cohorts. IL-6 was only measured in the HCP-CRHD dataset.

**Figure 3:**
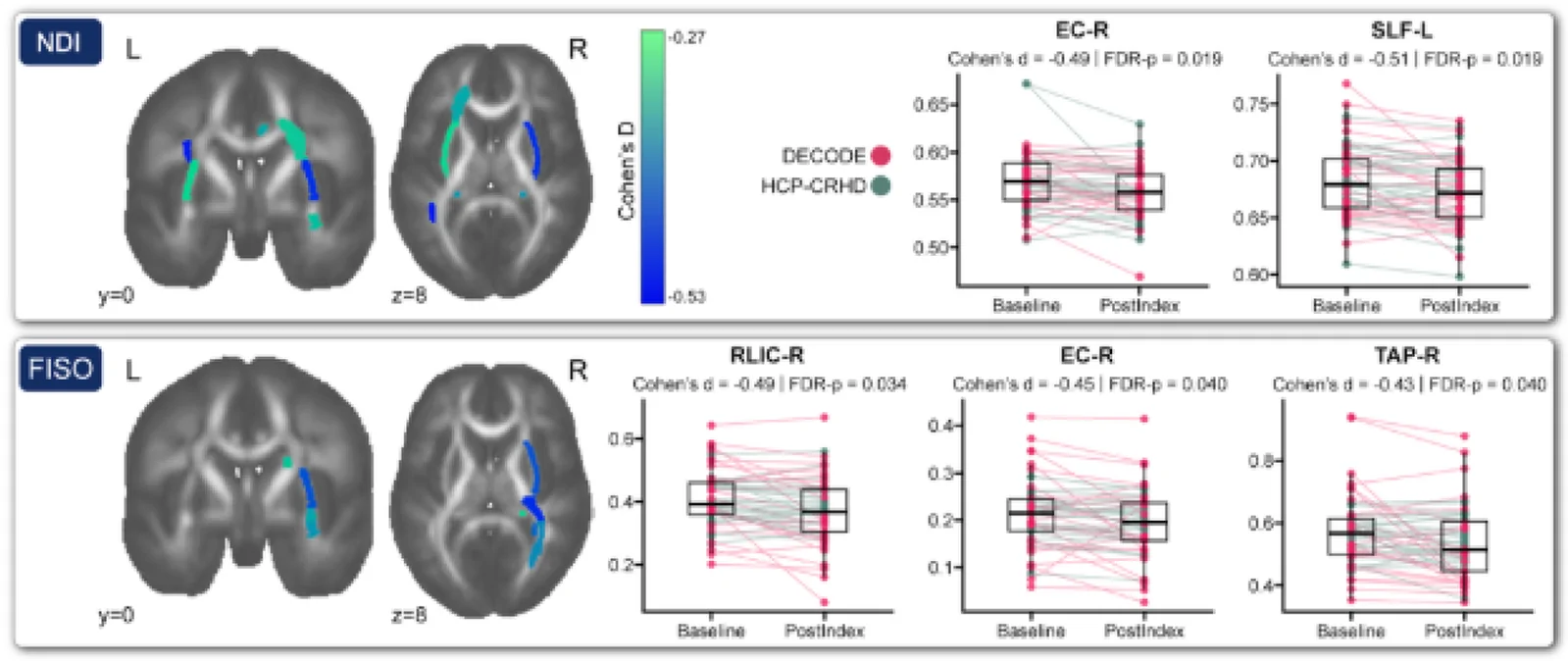
Standardized effect sizes are colorized and are plotted on all major WM tracts that exhibited FDR-corrected findings as well as trending significance (uncorrected p<0.05) showing overall decrease in NDI and FISO. NDI changes were observed in bilateral WM pathways, while FISO changes were only observed in the right hemisphere. Spaghetti plots for FDR-corrected significant regions are colored by study (EC-R= Right external capsule; SLF-L= left superior longitudinal fasciculus; RLIC-R= right retrolenticular part of internal capsule; TAP-R= right tapetum).

**Figure 4:**
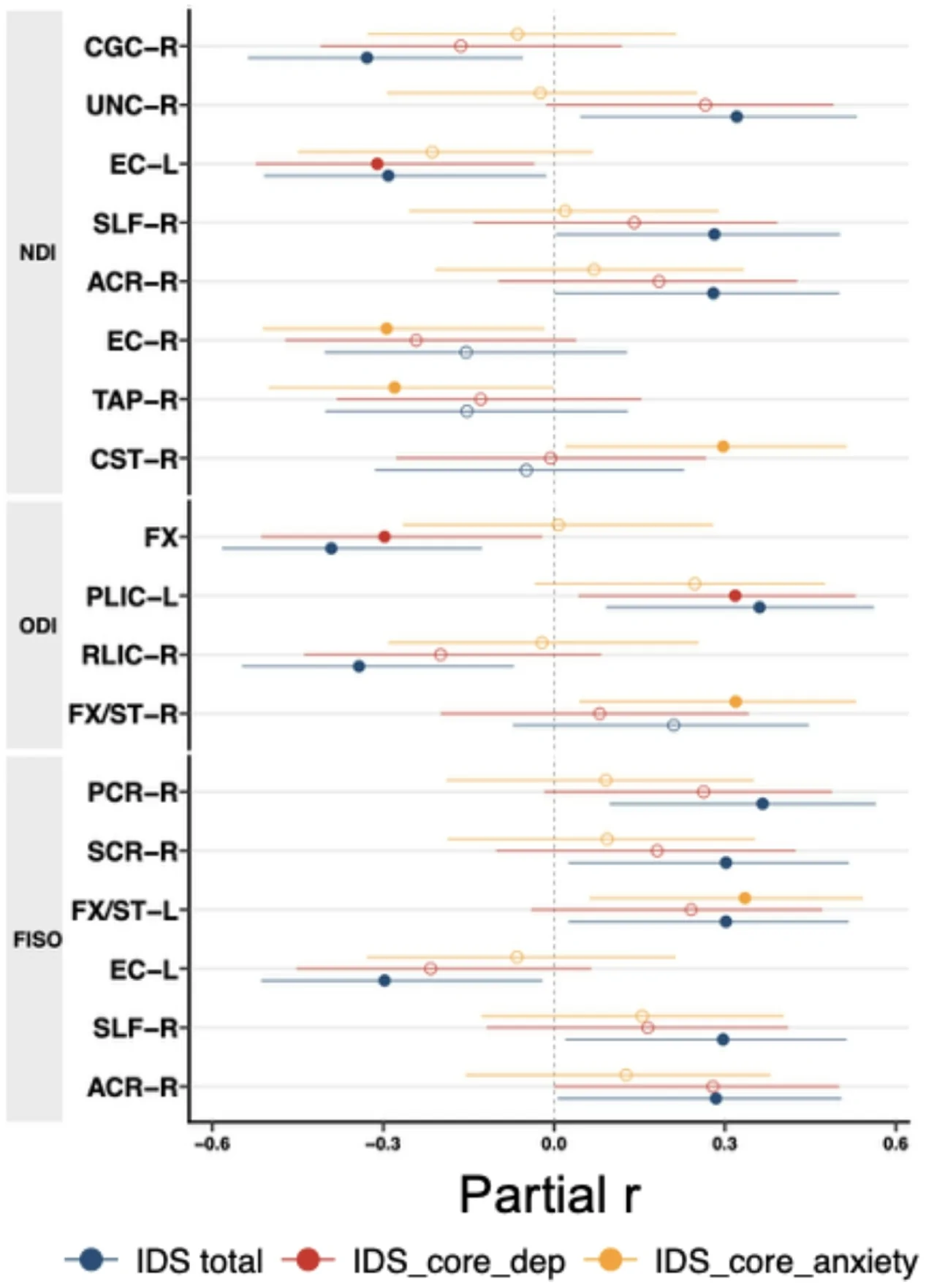
Associations between change in white-matter metrics and change in depressive symptoms for IDS-C total and subscales. For each ( tract ROI, diffusion metric) pair surviving p<0.05 in any of the three regressions including total IDS-C or core depression (IDS_core_dep), and core anxiety (IDS_core_anxiety) subscales, the partial r for the % Δ ROI predictor is shown with 95% confidence intervals. Filled circles indicate uncorrected p < 0.05; open circles indicate non-significant associations.

**Figure 5:**
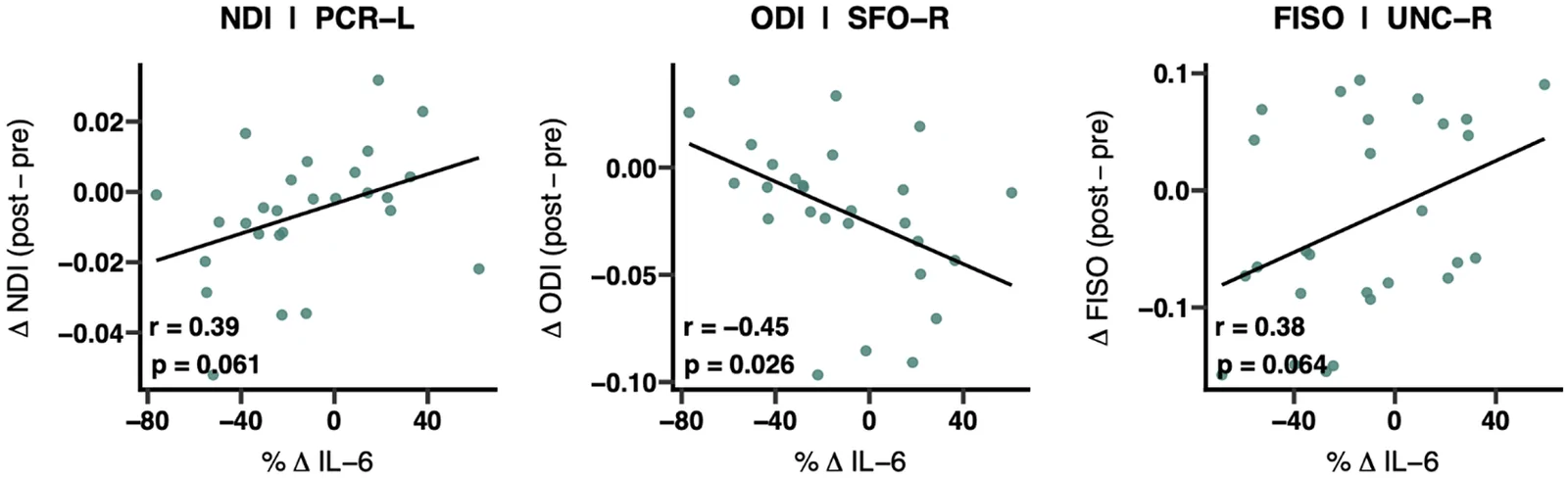
Scatter plots of pre-to-post-ECT change in ROI white-matter microstructure against percent change in IL-6 for the 7 ROIs reaching p < 0.065 in the per-region regression. Both axes are residualized for age, sex, and the baseline ROI value.

**Table 1:** Demographics for the merged sample as well as individual cohorts are listed. RUL= right unilateral; BT= bitemporal; BF= bifrontal; LART= left anterior right temporal. For additional demographic information see **Supplemental Table 3**.

Age (years)	DECODE (n = 24)	HCP-CRHD (n = 28)	Merged (n = 52)
	42.8 ± 17.0	37.7 ± 14.3	40.0 ± 15.7
Sex	62.5% Female	60.7%	61.5%
Baseline IDS	40.0 ± 8.2	41.2 ± 10.6	40.6 ± 9.5
% Change in IDS ± SD	−43.0% ± 26.2	−28.7 ± 25.5	−35.3 ± 26.5
Baseline to post-index			
Remitters	20.8% (n = 5)	10.7% (n = 3)	15.4% (n = 8)
Responders	41.7% (n = 10)	21.4% (n = 6)	30.8% (n = 16)
Number of ECT Sessions at Endpoint	11.4 ± 1.2	14.3 ± 5.2	12.9 ± 4.1
Electrode Placement	RUL = 23	RUL = 28	RUL = 51
(Baseline)	BF = 1		BF = 1
Electrode Placement	RUL = 8	RUL = 13	RUL = 21
(Endpoint/Post-Index)	BT = 15	BT = 13	BT = 28
	BF = 1	LART = 2	BF = 1
			LART = 2
Percentage RUL Post-Index	33.3%	46.4%	40.4%

**Table 2:** Effect of time in the pooled DECODE + HCP-CRHD sample. Regions showing effects at *p < 0.05* uncorrected are included for NDI, ODI, and FISO; the equivalent FA results are reported in Supplemental Table 4 and Supplemental Fig. 1. FDR-corrected results are marked in bold. The "Study p < 0.05" column reports whether the Time effect was significant when the analysis was repeated within each independent cohort alone (DECODE, HCP-CRHD, both, neither). β = Time coefficient; *t-val* = test statistic.

*White Matter Region*		Effect of Time					
	*Study p <* *0.05*	*β*	*SE*	*t-val*	*p-val*	*p-* *FDR*	*Cohen's* *D*
NDI							
**Superior longitudinal fasciculus left**	**Both**	**−0.01**	**0.003**	**−3.65**	**<.001**	**0.019**	**−0.51**
**External capsule right**	**Both**	**−0.01**	**0.003**	**−3.50**	**<.001**	**0.019**	**−0.49**
Fornix (cres) / Stria terminalis left	-	−0.01	0.003	−2.74	0.008	0.075	−0.38
Fornix (cres) / Stria terminalis right	HCP-CRHD	−0.01	0.005	−2.73	0.009	0.075	−0.38
Cingulum (cingulate gyrus) right	HCP-CRHD	−0.01	0.004	−2.62	0.012	0.075	−0.36
Anterior corona radiata left	HCP-CRHD	−0.01	0.003	−2.61	0.012	0.075	−0.36
Uncinate fasciculus right	HCP-CRHD	−0.02	0.010	−2.43	0.019	0.101	−0.34
Superior corona radiata right	-	0.00	0.002	−2.37	0.021	0.102	−0.33
External capsule left	HCP-CRHD	−0.01	0.005	−2.25	0.029	0.122	−0.31
Sagittal stratum left	HCP-CRHD	−0.01	0.003	−2.15	0.037	0.139	−0.30
ODI							
Fornix (column and body of fornix)	HCP-CRHD	−0.01	0.005	−2.53	0.015	0.352	−0.35
Uncinate fasciculus right	HCP-CRHD	−0.02	0.007	−2.35	0.023	0.352	−0.33
Inferior fronto-occipital fasciculus right	HCP-CRHD	−0.01	0.003	−2.27	0.028	0.352	−0.31
FISO							
**Retrolenticular part of internal capsule right**	**DECODE**	**−0.03**	**0.010**	**−3.53**	**<.001**	**0.034**	**−0.49**
**External capsule right**	**HCP-CRHD**	**−0.02**	**0.007**	**−3.22**	**0.002**	**0.040**	**−0.45**
**Tapetum right**	**DECODE**	**−0.04**	**0.012**	**−3.10**	**0.003**	**0.040**	**−0.43**
Posterior thalamic radiation right	-	−0.02	0.005	−2.82	0.007	0.063	−0.39
Uncinate fasciculus right	DECODE	−0.05	0.016	−2.75	0.008	0.063	−0.38
Inferior fronto-occipital fasciculus right	DECODE	−0.03	0.011	−2.63	0.011	0.072	−0.36
Superior fronto-occipital fasciculus right	HCP-CRHD	−0.02	0.008	−2.42	0.019	0.104	−0.34
Fornix (cres) / Stria terminalis right	HCP-CRHD	−0.04	0.016	−2.26	0.028	0.133	−0.31

Mixed-effects model: ROI ~ time + age + sex + study + (1 ∣ subject). FDR within metric.

## Data Availability

Neuroimaging and metadata for the HCP-CRHD cohort is available for download with the required Data Use Agreement through the NIH NDA (NDA, DOI: 10.15154/1528673). Neuroimaging and metadata for the DECODE project is being stored in the NDA and will be available for sharing with the scientific after the research has been completed (NDA Collection #4413).
